# Spatial variation in bidirectional pollinator-mediated interactions between two co-flowering species in serpentine plant communities

**DOI:** 10.1093/aobpla/plab069

**Published:** 2021-10-22

**Authors:** Aiden M. Stanley, Carlos Martel, Gerardo Arceo-Gómez

**Affiliations:** Department of Biological Sciences, East Tennessee State University, Johnson City, TN 37614, USA; Department of Biological Sciences, University of Pittsburgh, Pittsburgh, PA 15260, USA; Department of Biological Sciences, East Tennessee State University, Johnson City, TN 37614, USA; Instituto de Ciencias Ómicas y Biotecnología Aplicada, Pontificia Universidad Católica del Perú, San Miguel 15088, Lima, Peru; Department of Biological Sciences, East Tennessee State University, Johnson City, TN 37614, USA

**Keywords:** Competition, *Delphinium uliginosum*, facilitation, heterospecific pollen, *Mimulus guttatus*

## Abstract

Pollinator-mediated competition and facilitation are two important mechanisms mediating co-flowering community assembly. Experimental studies, however, have mostly focused on evaluating outcomes for a single interacting partner at a single location. Studies that evaluate spatial variation in the bidirectional effects between co-flowering species are necessary if we aim to advance our understanding of the processes that mediate species coexistence in diverse co-flowering communities. Here, we examine geographic variation (i.e. at landscape level) in bidirectional pollinator-mediated effects between co-flowering *Mimulus guttatus* and *Delphinium uliginosum.* We evaluated effects on pollen transfer dynamics (conspecific and heterospecific pollen deposition) and plant reproductive success. We found evidence of asymmetrical effects (one species is disrupted and the other one is facilitated) but the effects were highly dependent on geographical location. Furthermore, effects on pollen transfer dynamics did not always translate to effects on overall plant reproductive success (i.e. pollen tube growth) highlighting the importance of evaluating effects at multiple stages of the pollination process. Overall, our results provide evidence of a spatial mosaic of pollinator-mediated interactions between co-flowering species and suggest that community assembly processes could result from competition and facilitation acting simultaneously. Our study highlights the importance of experimental studies that evaluate the prevalence of competitive and facilitative interactions in the field, and that expand across a wide geographical context, in order to more fully understand the mechanisms that shape plant communities in nature.

## Teaser

Pollinator-mediated interactions among co-flowering plants can result in positive (facilitation), negative (competition) or neutral outcomes for plants. Despite their importance in plant community assembly, experimental assessment of the bidirectional effects of pollinator-mediated interactions is still limited. Based on evaluation of pollen transfer dynamics (conspecific and heterospecific pollen deposition) and plant reproductive success of co-flowering *Mimulus guttatus* and *Delphinium uliginosum*, we show asymmetrical pollinator-mediated effects which were dependent on geographical location. Our results show that competition and facilitation can act simultaneously between co-flowering species and provide evidence of a spatial mosaic of pollinator-mediated interactions.

## Introduction

It has been widely demonstrated that pollinator-mediated interactions between co-flowering plants can have both direct (e.g. improper pollen transfer; Morales and Traveset 2008; Ashman and Arceo-Gómez 2013) and indirect effects (e.g. via pollinator visitation; Mitchell *et al.* 2009; Losapio and Schöb 2020) on plant reproductive success. These interactions can in turn have important ecological and evolutionary consequences and influence floral evolution and co-flowering community assembly (Galen 1999; Kay and Sargent 2009; Mitchell *et al.* 2009; Ashman and Arceo-Gómez 2013; Carvalheiro *et al.* 2014). Among these, two main types of indirect plant–plant interactions via changes in pollinator preference have been described, i.e. pollinator competition and facilitation (Campbell 1985; Brown *et al.* 2002; Feldman *et al.* 2004; Ghazoul 2006; Mitchell *et al.* 2009). Competition occurs when the presence of one species decreases pollinator visitation to another (e.g. Campbell 1985; Brown *et al.* 2002; Mitchell *et al.* 2009), negatively affecting the pollination success of one or both species; whereas in facilitation pollinator visitation increases for at least one plant species when two or more species flower simultaneously (e.g. Feldman *et al.* 2004; Ghazoul 2006; Carvalheiro *et al.* 2014) leading to an increase in pollination success. Thus, pollinator-mediated interactions between two plants species can result in six different scenarios, i.e. positive outcomes for both partners (+:+), positive for one and negative for the other (+:−), positive and neutral (+:0), negative and neutral (−:0) and negative (−:−) or neutral (0:0) for both partners. Pollinator-mediated interactions however have historically been studied by focusing on only one of the interacting partners (e.g. Campbell 1985; Morales and Traveset 2008; Flanagan *et al.* 2009; Muchhala *et al.* 2009; Arceo-Gómez and Ashman 2011; but see Caruso and Alfaro 2000; Ghazoul 2006; Muchhala *et al.* 2010; Suárez-Mariño *et al.* 2019; Streher *et al.* 2020; Bergamo *et al.* 2020b; Ha *et al.* 2021). Although central in advancing our understanding of the drivers and consequences of pollinator-mediated interactions, studies that focus on only one interacting partner may underestimate the real complexity of these interactions in nature. Specifically, a unidirectional focus overlooks the potential for bidirectional effects between individual species pairs (e.g. Bascompte and Jordano 2007; Vila *et al.* 2009), which can further influence floral evolution and community assembly. In this sense, plant species pairs can experience either similar (both impair or facilitate each other) or asymmetrical effects (e.g. one experiences competition while the other one is facilitated; Caruso and Alfaro 2000; Ghazoul 2006; Muchhala *et al.* 2010; Suárez-Mariño *et al.* 2019). It is also possible that one species will be affected (positively or negatively) while its interacting partner will not (neutral effect). However, the occurrence and directionality of these two-sided interactions have surprisingly received considerably less attention compared to the study of unidirectional effects. Studies that consider bidirectional effects between co-flowering species are necessary if we aim to advance our understanding of the processes that mediate plant species coexistence in diverse co-flowering communities.

The strength and direction of pollinator-mediated interactions can vary spatially due to changes in the composition of the surrounding plant and pollinator community, potentially leading to geographic mosaics in the outcomes of species interactions (e.g. Thompson 1999, 2005; Moeller 2005). For example, changes in pollinators’ availability or co-flowering community composition can influence patterns of pollinator-mediated facilitation and competition (Anderson and Johnson 2007; Lázaro *et al.* 2009; Martén-Rodríguez *et al.* 2011). However, very few studies have evaluated geographic variation in the outcomes of pollinator-mediated interactions between co-flowering species (Olesen and Jordano 2002; Burkle *et al.* 2016). It is thus imperative that we fully evaluate the extent of spatial variation in the outcome of pollinator-mediated interactions and how this may lead to spatial mosaics of floral evolution and community assembly (Thompson *et al.* 1999, 2005). Finally, the strength and direction of pollinator-mediated plant–plant interactions have often been determined by evaluating effects on patterns of pollinator visitation alone (e.g. Waser *et al.* 1983; Feldman *et al.* 2004; Mitchell *et al.* 2009). However, studies have shown that pollinator visitation may not accurately reflect pollination success (King *et al.* 2013; Ballantyne *et al.* 2015; Stavert *et al.* 2016), mainly because pollinators vary in their ability to successfully transfer and deposit conspecific pollen (Benevides *et al.* 2013; King *et al.* 2013; Stavert *et al.* 2016). Pollinators may also vary in the quality of pollen they transport (conspecific vs. heterospecific pollen; Morales and Traveset 2008; Ashman *et al.* 2020). Heterospecific pollen transfer has been shown to impose strong negative effects on males (Muchhala *et al.* 2010) and female reproductive success (Ashman and Arceo-Gómez 2013), potentially strengthening the effects of pollinator competition or offsetting the effects of facilitation via increased pollinator visitation. Thus, heterospecific pollen transfer may have the potential to affect the directionality of pollinator-mediated interactions among co-flowering plants. For instance, the presence of one plant species may lead to increased visitation to another species (i.e. reproductive facilitation via increased visitation) but also lead to an increase in heterospecific pollen (i.e. reproductive competition via pollen interference) and the overall outcome of the interaction may be negative (e.g. Morales and Traveset 2008; Thomson *et al.* 2019). Thus, studies that evaluate the strength and direction of pollinator-mediated interactions by integrating multiple aspects of the pollination process including conspecific and heterospecific pollen deposition as well as pollination success (e.g. pollen tube growth) are key in order to fully evaluate the consequences of these interactions in nature.

**Figure 1.**
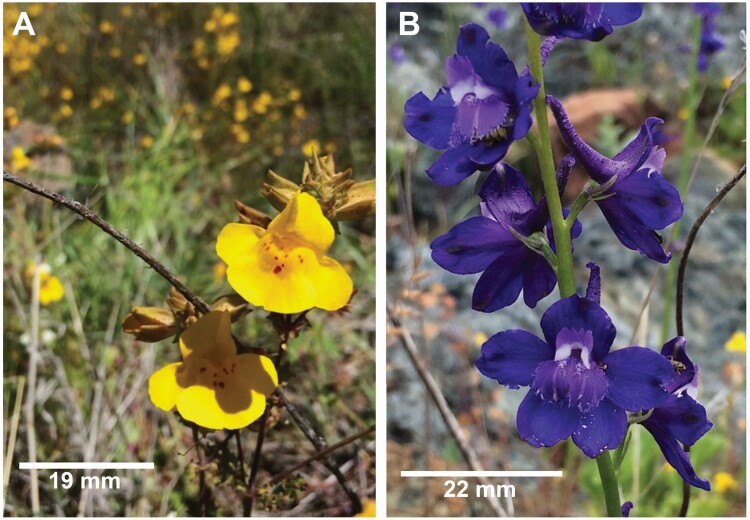
Studied plant species. (A) *Mimulus guttatus* and (B) *Delphinium uliginosum.*

In this study, we examine geographical variation in the bidirectional effects of pollinator-mediated interactions between two co-flowering species, *Mimulus guttatus* (Phrymaceae) and *Delphinium uliginosum* (Ranunculaceae). We evaluated these effects across multiple stages of the pollination process (i.e. conspecific and heterospecific pollen deposition, pollen tube growth). These two species differ widely in their floral phenotypes (Fig. 1). Furthermore, *M. guttatus* is typically more abundant and considered a pollinator generalist while *D. uliginosum* tends to be primarily bumblebee-pollinated, which also visits *M. guttatus* flowers (Koski *et al.* 2015). Thus, it is possible to expect that *M. guttatus* may help facilitate pollination success in the less abundant *D. uliginosum* (Wei *et al.* 2021). However, this may occur at the detriment of *M. guttatus* pollination, either via a decrease in conspecific pollen or via an increase in heterospecific pollen deposition (Wei *et al.* 2021). Specifically, we ask the following questions: (i) do patterns of conspecific and heterospecific pollen deposition, and pollen tube growth differ between *M. guttatus* and *D. uliginosum* when they grow alone compared to when they occur together? (ii) is the outcome of species pair interactions the same or asymmetrical? and (iii) does the outcome of these interactions vary across three different sites?

## Materials and Methods

### Focal species

We use *M. guttatus* as a focal species because it is a key species mediating indirect pollinator interactions in the serpentine seep co-flowering communities in northern California (Koski *et al.* 2015). Due to its long flowering period and high floral abundance *M*. *guttatus* has the potential to engage in positive and negative pollinator-mediated interactions with a wide array of co-flowering species across the season (Koski *et al.* 2015). *Mimulus guttatus* (MIGU) produces yellow zygomorphic flowers (Fig. 1) that are predominantly pollinated by bumblebees but also attract a variety of other pollinators including solitary bees, beetles, butterflies and flies (Arceo-Gómez and Ashman 2014a; Koski *et al.* 2015). We specifically evaluated how *M. guttatus* interacted with *D. uliginosum* via pollinators. We chose *D. uliginosum* due to its high abundance at the seeps and its high pollinator use overlap with *M. guttatus* (Koski *et al.* 2015) despite strong differences in flower morphology (i.e. size, shape and colour; Fig. 1). *Delphinium uliginosum* (DEUL) is a perennial herb that produces blue-purple flowers that are primarily pollinated by bumblebees at the study sites, but plants are also visited by butterflies and other bees (Koontz and Warnock 2012; Koski *et al.* 2015; G. Arceo-Gómez, unpubl. data; Fig. 1).

### Study sites

This study was conducted at three locations (i.e. serpentine seeps; here identified as BS, RHA, TP9) at the McLaughlin Natural Reserve in Lower Lake California, USA (38°52′16.2″N, 122°25′09.5″W). The seeps are separated by an average of 2.01 ± 0.46 km (mean ± SD) and are surrounded by vast swathes of grassland, small shrubs and chaparral species. Seep communities are typically composed of a mix of annual and perennial animal-pollinated species. Species richness at the seeps can vary from only a few to more than 30 plant species (Arceo-Gómez and Ashman 2014a; Koski *et al.* 2015). Serpentine seeps are ecologically similar in that they share similar environmental conditions and unique soil chemistry and have thus been considered as replicates of similar plant–pollinator communities within the same geographic area (Freestone and Harrison 2006; Arceo-Gómez and Ashman 2014a; Arceo-Gómez *et al.* 2016, 2018). Our sites were selected based on the presence of both focal species and were composed of a core of 39–62 species. Each site was visited twice per week between 16 and 31 May in 2018 (five total visits per site), which falls within the flowering peak of the studied species (Arceo-Gómez *et al.* 2018). At each site, we delineated 1 × 2 m plots ensuring plots contained one of three species composition treatments: (i) *M. guttatus* alone (MIGU only), (ii) a mix of *M. guttatus* and *D. uliginosum* (DEUL–MIGU) and (iii) *D. uliginosum* alone (DEUL only). We established 5–7 plots (separated by at least 5 m) for each species composition treatment per seep for a total of 17–19 plots per treatment across all three seeps (BS: 5 DEUL only, 6 MIGU only, 6 DEUL–MIGU; RHA: 7 DEUL only, 6 MIGU only, 6 DEUL–MIGU; TP9: 6 DEUL only, 6 MIGU only, 6 DEUL–MIGU). Although plots sometimes contained individuals of other plant species in the community ~90 % of flowers (average 138.4 flowers per plot) within the plot belonged to the focal species studied.

### Pollen transfer dynamics and post-pollination success

To evaluate differences in pollen transfer dynamics and pollen tube growth between plants in different plot treatments we collected three styles per species per plot during each visit to a site, (each style from a different individual). We collected styles from flowers that were at the end of their lifetime (wilted flowers) and thus were no longer visited. In total we collected 172–195 *M*. *guttatus* styles (87–90 single and 85–102 mixed plots), and 89–166 styles of *D. uliginosum* (48–96 single and 41–71 mixed plots) across all three seeps. Styles were stored in 70 % ethanol until processing and softened and stained with aniline blue using standard methods in the laboratory (Dafni 1992; Arceo-Gómez *et al.* 2016). We collected data for a total of 950 flowers (539 of *M. guttatus* and 411 of *D. uliginosum*).

Pollen loads on the stigma were visualized using a compound microscope at 400× magnification. We recorded the total number of conspecific pollen (pollen belonging to the recipient species) and heterospecific pollen (pollen belonging to a different species than the recipient). We did not limit our heterospecific pollen counts to only our focal plant species, but that from any other co-flowering plant species (focal and non-focal). To aid in the identification of pollen grains as either *M. guttatus* or *D. uliginosum*, we previously created a pollen reference library by collecting pollen from mature anthers from both species in the field. A total of 178 276 pollen grains were counted across the two species and the three seeps.

We also counted the number of pollen tubes that reached the base of the style under a fluorescent microscope. The number of pollen tubes has been considered a good proxy of pollination success (e.g. Alonso *et al.* 2012; Arceo-Gómez and Ashman 2014b). We performed this for a subset of randomly selected slides from each treatment group. A total of 50 *M. guttatus* (25 from mixed, 25 from single plots), and 50 *D. uliginosum* (25 from mixed, 25 from single plots) slides were counted. *Delphinium uliginosum* flowers have 1–3 styles and thus conspecific pollen loads, heterospecific pollen loads and pollen tubes were counted on all styles and added.

### Data analysis

To evaluate the strength and direction of pollinator-mediated interactions between the two co-flowering species (i.e. *M. guttatus* and *D. uliginosum*), we ran three separate analyses. We conducted generalized linear mixed models to evaluate differences in conspecific pollen loads, heterospecific pollen loads and the proportion of pollen tubes that reached the base of the style (i.e. number of pollen tubes/total conspecific pollen load). Evaluating differences in conspecific and heterospecific pollen loads separately allows evaluating the relative importance of the two main mechanisms by which plants interact with each other via pollinators, i.e. via pollinator visitation (hence altering conspecific pollen receipt; Mitchell *et al.* 2009) and via heterospecific pollen interference on the stigma (Ashman and Arceo-Gómez 2013). Further, the evaluation of the proportion of pollen tubes reflects the effect of pollinator-mediated interactions on overall female fitness. For all analyses we used plot species composition treatment (i.e. alone vs. mixed), site and plant species as fixed effects. Plot ID was set as a random factor. Because we were also interested in evaluating whether responses between alone versus mixed treatments vary by site, species or both, we also included the site * treatment, treatment * species and the three-way interaction as fixed effects. We used negative binomial distribution with log link function for conspecific and heterospecific pollen loads and a binomial distribution with logit link function for the proportion of pollen tubes. When a three-way interaction was significant, we used *a priori* contrasts (see Arceo-Gómez and Ashman 2014a) to specifically evaluate differences between single and mixed plot treatments for each species at each one of the three seeps (e.g. Molofsky and Fisher 1993; Rosenthal *et al.* 2000; Strauss and Murch 2004; Arceo-Gómez and Ashman 2014a). All flowers within a plot (flower density) were counted and used as a covariate in the analyses. Model fit (overdispersion) was evaluated using the residual deviance against the degrees of freedom of the model; this resulted in values close to one, which indicates that our models were not overdispersed. All analyses were conducted using the *lme4* (Bates *et al.* 2019), *MASS* (Ripley *et al.* 2020) and *emmeans* packages (Lenth *et al.* 2020) in R (R version 3.6.1; R Development Core Team 2017).

## Results

### Conspecific pollen deposition

**Figure 2.**
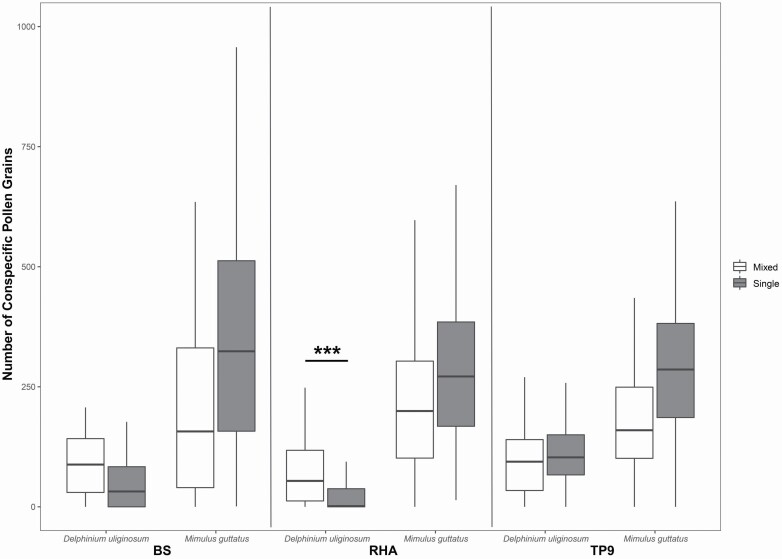
Number (mean ± SE) of conspecific pollen grains found on styles by *D. uliginosum* and *M*. *guttatus* in single or mixed plots at three different sites (BS, RHA, TP9). Significant *post hoc* comparisons among species * site * treatment interactions are shown. ***P < 0.001.

Average conspecific pollen load was significantly higher in *M. guttatus* (mean ± SD: 262.8 ± 9.1 pollen grains) compared to *D. uliginosum* (mean ± SD: 73.8 ± 3.5 pollen grains; χ ^2^ = 255.70, df = 1, *P* < 0.001). This however is not surprising since the former produces far more ovules (~400 seeds; Arceo-Gómez and Ashman 2014a) compared to the latter (typically less than 50; G. Arceo-Gómez, pers. obs.). Conspecific pollen load size also differed significantly among sites (χ ^2^ = 19.71, df = 2, *P* < 0.001), but not between species composition treatments (single vs. mixed; χ ^2^ = 0.18, df = 1, *P* = 0.67). However, we found a significant site * treatment (χ ^2^ = 13.1, df = 2, *P* = 0.001), treatment * species (χ ^2^ = 18.36, df = 1, *P* < 0.001) and a marginally significant site * treatment * species interaction (χ ^2^ = 5.34, df = 2, *P* < 0.07; Fig. 2). *A priori* contrasts showed that *D. uliginosum* received significantly more conspecific pollen when growing with *M. guttatus* (MIGU–DEUL plot) compared to when growing alone (DEUL-only plot), although this difference was only significant at one site (i.e. RHA; *P* < 0.001 for both; Fig. 2). On the other hand, *M. guttatus* tended to receive larger conspecific pollen loads when growing alone (single MIGU plots) although these differences were not significant (*P* > 0.05 for all; Fig. 2). Floral density had a positive significant effect on conspecific pollen receipt (χ ^2^ = 5.81, df = 1, *P* < 0.05).

### Heterospecific pollen deposition

**Figure 3.**
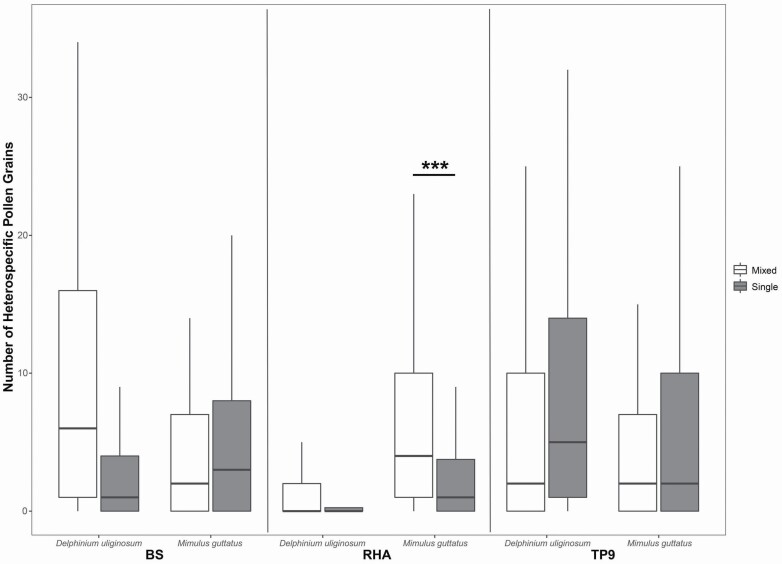
Number (mean ± SE) of heterospecific pollen grains found on styles of *D*. *uliginosum* and *M*. *guttatus* in single or mixed plots at three different sites (BS, RHA, TP9). Significant *post hoc* comparisons among species * site * treatment interactions are shown. ***P < 0.001.

Average heterospecific pollen load size differed significantly between *M. guttatus* (mean ± SD: 7.41 ± 0.72 heterospecific pollen grains) and *D*. *uliginosum* (mean ± SD: 5.50 ± 0.58; χ ^2^ = 12.07, df = 1, *P* < 0.001), among sites (χ ^2^ = 40.88, df = 2, *P* < 0.001), and was marginally significant between plot species composition treatments (single vs. mixed; χ ^2^ = 3.20, df = 1, *P* = 0.07). We also found a significant site * treatment interaction (χ ^2^ = 9.44, df = 2, *P* < 0.01), but the treatment * species interaction was not significant (χ ^2^ = 0.23, df = 1, *P* = 0.63). However, the site * treatment * species interaction was significant (χ ^2^ = 14.94, df = 2, *P* < 0.001; Fig. 3). *A priori* contrasts showed that *M*. *guttatus* receives significantly larger amounts of heterospecific pollen when growing in mixed (MIGU–DEUL) compared to MIGU-only plots, but only at one site (i.e. RHA, *P* < 0.001; Fig. 3). Single versus mixed plot differences at the remaining two sites were not significant (*P* > 0.05; Fig. 3). We were unable to detect differences in heterospecific pollen load size between *D. uliginosum* plants growing in single versus mixed plots across all sites (*P* > 0.05 for all; Fig. 3). Floral density did not affect heterospecific pollen load size (χ ^2^ = 1.43, df = 1, *P* = 0.23).

### Pollen tube growth

**Figure 4.**
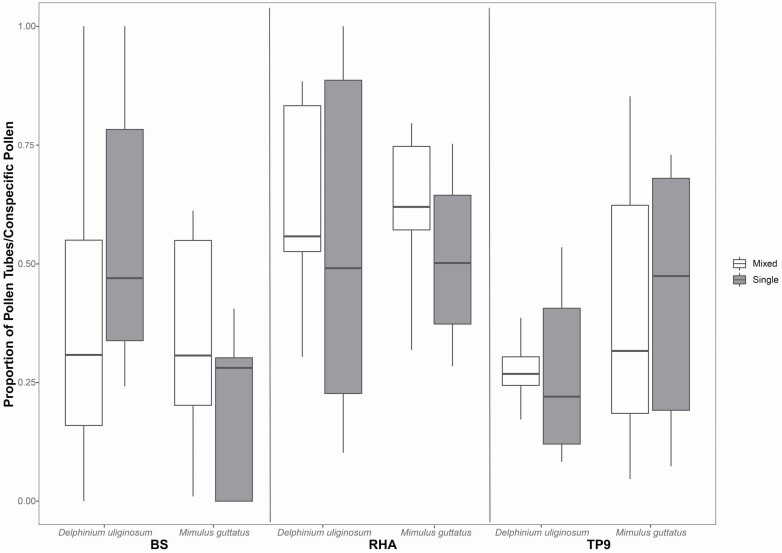
Proportion of pollen tubes/conspecific pollen found on styles of *D*. *uliginosum* and *M. guttatus* in single or mixed plots. *Post hoc* comparisons between species * site * treatment interactions were not significant.

The proportion of pollen tubes (pollen tubes/conspecific pollen load) did not differ significantly between *M. guttatus* (mean ± SD: 0.40 ± 0.04) and *D. uliginosum* (mean ± SD: 0.26 ± 0.24; χ ^2^ = 1.05, df = 1, *P* = 0.31) or between plot species composition treatments (alone vs. mixed; χ ^2^ = 0.76, df = 1, *P* = 0.38). Although we identified differences among sites (χ ^2^ = 7.71, df = 2, *P* < 0.05), we did not find significant effects of the site * treatment (χ ^2^ = 3.20, df = 2, *P* = 0.20), site * species (χ ^2^ = 1.27, df = 2, *P* = 0.53), treatment * species (χ ^2^ = 0.67, df = 1, *P* = 0.41) or site * treatment * species interactions (χ ^2^ = 0.70, df = 2, *P* = 0.70) on the proportion of pollen tubes (Fig. 4). Floral density did not have an effect on the proportion of pollen tubes (χ ^2^ = 0.01, df = 1, *P* = 0.92).

## Discussion

Evaluating the processes that mediate plant species coexistence is central for developing a predictive understanding of the mechanisms that govern the assembly of plant communities and how these may change in response to human disturbances (Sargent and Ackerly 2008; Elo *et al.* 2016). In this sense, pollinator competition and facilitation have been commonly identified as the most important single-acting forces in the organization of co-flowering communities at a local scale (after dispersal and environmental filters; Levine 1999; Choler *et al.* 2001; Callaway *et al.* 2002; Brooker 2006; Brooker *et al.* 2008; Mitchell *et al.* 2009). Our results, however, provide evidence suggesting a more complex mosaic of pollinator-mediated interactions and that community assembly processes could result from both types of interaction (i.e. competition and facilitation) acting together rather than singly. Specifically, we show evidence of bidirectional interactions between plant species with asymmetrical outcomes that are highly dependent on the site where they occur. Experiments that evaluate the prevalence of these interactions in the field, and that expand across a wide geographical context, are necessary to more fully understand the mechanisms that shape plant communities in nature.

The distribution of plant functional traits within a community has been increasingly used to infer the processes that govern plant community assembly (Westoby and Wright 2006; Kraft and Ackerly 2014; Xu *et al.* 2018; Navarro-Cano *et al.* 2021), including those mediated by pollinator-mediated interactions (e.g. Sargent and Ackerly 2008; McEwen and Vamosi 2010; de Jager *et al.* 2011; Wolowski *et al.* 2017; Bergamo *et al.* 2018). However, underlying this approach is the implicit assumption that either competition or facilitation is singly responsible for structuring co-flowering communities. Here we show evidence that both competition and facilitation could act simultaneously. Specifically, *M. guttatus* tended to receive less conspecific pollen (although not significantly; Fig. 2) and more heterospecific pollen (e.g. at RHA; Fig. 3) when co-flowering with *D. uliginosum*. These results are consistent with both forms of pollinator-mediated competition, i.e. via changes in pollinator visitation and via heterospecific pollen interference. On the contrary, *D. uliginosum* received significantly more conspecific pollen when co-flowering with *M. guttatus* (at RHA; Fig. 2) thus indicating that it may benefit from the presence of *M. guttatus*, at least at some sites (see below). Interestingly, plant–pollinator network studies have long proposed the existence of strong asymmetries in the strength and potential effects of species interactions (Jordano 1987; Vázquez and Aizén 2004; Bascompte *et al.* 2006). Therefore, it is somewhat surprising that these asymmetries, and their role in community assembly, have not been more often considered when evaluating community-wide consequences of pollinator-mediated interactions. For instance, recent studies that have evaluated the role of phenotypic traits in determining the outcome of community-wide species interactions have also suggested that both, competition and facilitation, likely play a role in the assembly of plant communities (Tur *et al.* 2016; Bergamo *et al.* 2018; Navarro-Cano *et al.* 2021; Wei *et al.* 2021). Although the importance of the interplay between competition and facilitation in the assembly of co-flowering communities was proposed more than 35 years ago (Rathcke 1983; Mitchell *et al.* 2009), only few studies have experimentally evaluated the bidirectionality of pollinator-mediated interactions between co-flowering species (i.e. Randall and Hilu 1990; Rahmé *et al.* 2009; Sieber *et al.* 2011; Briscoe Runquist 2012; Briscoe Runquist and Stanton 2013; Wassink and Caruso 2013). We thus emphasize the need for experimental studies that evaluate the directionality of pollinator-mediated interactions in the field in order to validate predictions resulting from trait-based assembly and network studies. Such studies would also help to develop a more predictive understanding of the role of competition and facilitation in structuring plant communities.

In our study, the asymmetrical outcome in patterns of pollen receipt observed between *M. guttatus* and *D*. *uliginosum* may be the result of differences in several floral characteristics. For instance, *D*. *uliginosum* flowers have large amounts of nectar while *M. guttatus* flowers offer little to no nectar at our study sites (Robertson *et al.* 1994; G. Arceo-Gómez, pers. obs.). Hence, bumblebees (primary floral visitors) may be highly attracted to mixed-species plots but preferentially visit the resource rich *D*. *uliginosum* flowers, to the detriment of *M. guttatus* flowers, thus generating positive effects for the former and negative effects for the latter. Furthermore, *M. guttatus* flowers are more generalized and also visited by other insects such as beetles, flies and butterflies (Koski *et al.* 2015; Wei *et al.* 2021), which may be more abundant in mixed-species plots but are be less efficient and deposit higher amounts of heterospecific pollen loads. On the contrary, *D*. *uliginosum* is more specialized and primarily visited by bumblebees that feed on nectar-rich resources, which may result in higher visitor efficiency and fidelity. In fact, bumblebee visitation was more than three times higher for *D*. *uliginosum* flowers (0.007 visits per flower per min) than for *M. guttatus* (0.002 visits per flower per min; G. Arceo-Gómez, unpubl. data). *Mimulus guttatus* in turn receives a higher visitation from other insects in the community (0.017 visits per flower per min) compared to *D*. *uliginosum* (0.005 visits per flower per min; G. Arceo-Gómez, unpubl. data). Moreover, *M. guttatus* is a dominant species (70 flowers per m^2^) in the studied seep communities, particularly compared to *D*. *uliginosum* (32 flowers per m^2^; G. Arceo-Gómez, unpubl. data; also see Koski *et al.* 2015). Abundant species have been proposed to be more prone to competitive interactions while less abundant and more specialized species are often facilitated (Ghazoul 2005, 2006; Bizecki Robson 2013; Zografou *et al.* 2020; Wei *et al.* 2021). Overall, these results highlight the dynamic nature of pollinator-mediated interactions (e.g. Rathcke 1983; Feinsinger 1987; Tur *et al.* 2016), and suggest that their effects can be determined by plant species pollination niches (de Jager *et al.* 2011; Phillips *et al.* 2020; Wei *et al.* 2021). However, the exact mechanisms mediating asymmetrical interactions were not evaluated here and studies that evaluate the factors mediating these asymmetries across plant species pairs are needed in order to fully understand the processes that shape natural plant communities.

Similar to other ecological interactions, the outcomes of pollinator-mediated interactions can vary along species’ distribution range, with potential implications on floral diversification (Thompson 1999, 2005; Gómez *et al.* 2009b; Thompson *et al.* 2017; Friberg *et al.* 2019) and community assembly (Briscoe-Runquist *et al*. 2016; Tur *et al.* 2016). In spite of this, studies that evaluate plant–plant interactions via pollinators have been typically conducted at a single location (e.g. Ye *et al.* 2014; Ha and Ivey 2017; Bergamo *et al.* 2020a, b; Ha *et al.* 2021), thus neglecting the importance of geographic differences in the outcome of these interactions (but see Sieber *et al.* 2011). For instance, our results show that *M. guttatus* receives less heterospecific pollen when growing alone, but these effects were not significant at all sites. Similarly, *D. uliginosum* conspecific pollen loads were significantly higher when growing in mixed plots, but only at one site. Interestingly, both of these significant differences were observed at the same site (RHA; Figs 2 and 3), which also had the largest floral density compared to the two other sites (BS: 136.9, RHA: 161.1, TP9: 115.5 flowers per m^2^). Thus, it is possible that the outcome of pollinator-mediated interactions in these communities is density-dependent (also see Wei *et al.* 2021). Overall, our results thus suggest that the occurrence of competitive or facilitative interactions, even between the same plant species pair, is highly context-dependent. These differential responses might be explained by differences in plant richness among sites (BS: 62, RHA: 47, TP9: 39 plant species; Arceo-Gómez *et al.* 2018), differences in pollinator assemblages (Koski *et al.* 2015) and/or due to changes in specific pollinator preferences among the study sites (Ellis *et al.* 2021). Geographic variation in the outcome of these interactions can also result from changes in the number and identity of the pollinator assemblage (Gómez *et al.* 2009a) and plant community composition, as it is also exemplified in studies of diffuse selection (reviewed in Strauss *et al.* 2005). For instance, a study at the same study sites showed that *M. guttatus* pollen limitation (e.g. pollinator competition) depends strongly on the species richness of the surrounding co-flowering community (Arceo-Gómez and Ashman 2014b). Thus, the outcome of a specific plant–plant interaction at a single location may not reflect how the same plant species interact along their entire distribution range. The differential outcome of these interactions across spatial scales could have important implications for community assembly as they would favour coexistence and co-flowering in some sites while discouraging it in others (e.g. Briscoe-Runquist and Stanton 2013). Spatial variation in the outcome of these interactions can also contribute to generating geographical mosaics of selection (Thompson 1999, 2005), as the relationship between floral traits and fitness would vary spatially with varying intensity of competition and facilitation. For instance, Siebert *et al.* (2011) reported no evidence for either competition or facilitation at small spatial scales but they did detect facilitation at large scales. To our knowledge, this study is one of few (see also Sieber *et al.* 2011) to show geographical variation in the outcome of bidirectional pollinator-mediated interactions. Thus, studies that evaluate facilitative and competitive pollinator-mediated interactions across large spatial scales are needed in order to understand their contribution to floral evolution and plant community assembly.

Finally, studies of pollinator-mediated interactions have almost exclusively relied on estimates of flower visitation to infer positive versus negative interactions among plant species (e.g. Ghazoul 2006; Sieber *et al.* 2011; Ye *et al.* 2014; van der Kooi *et al.* 2016) despite that flower visitation alone can be a poor proxy of overall pollination success (e.g. King *et al.* 2013). In fact, recent studies have shown that flower visitation alone is likely insufficient to capture the full complexity of the pollination and fertilization process that more directly relates to plant reproductive success (Ashman *et al.* 2020). For example, although heterospecific pollen loads on styles are rather low compared to conspecific pollen loads, which has also been reported for most studied plants (Arceo-Gómez *et al.* 2019), and differences in heterospecific pollen deposition are not high among the experimental plots, the small loads and differences of heterospecific pollen do not prevent their potential negative effects on plant reproduction. Thus, estimates of conspecific pollen (i.e. pollen limitation) and heterospecific pollen (i.e. pollen interference) receipt, along with estimates of plant fitness (i.e. pollen tube growth, seed production), are necessary in order to more accurately describe the outcomes and underlying mechanisms driving plant–plant interactions via pollinators (e.g. Tur *et al.* 2016; Ashman *et al.* 2020). For instance, while we observed a significant increase in *D. uliginosum* conspecific pollen receipt when co-flowering with *M. guttatus* (at one site), this increase did not affect ovule fertilization success (i.e. pollen tube growth). This could be because *D. uliginosum* is not pollen-limited at the study sites and therefore all plants have received enough conspecific pollen to maximize pollen tube production. Similarly, an increase in *M. guttatus* heterospecific pollen receipt in mixed plots compared to single plots (at one site) did not translate to a decrease in *M. guttatus* reproductive success; perhaps due to high *M. guttatus* tolerance to interference by *D. uliginosum* pollen (perhaps due to their relatively large phylogenetic distance; Ashman and Arceo-Gómez 2013; Streher *et al.* 2020). What is evident, however, is that in order to accurately describe the outcomes and identify the underlying mechanisms mediating pollinator-mediated interactions, it is necessary to evaluate their effects at multiple stages of the pollination process, i.e. from pollen deposition to ovule fertilization success.

## Data Availability

All data used for analysis in this publication can be found in the following Dryad repository (doi:10.5061/dryad.nzs7h44sm).

## Notes

**Populations & Communities. Chief Editor:** Jean Burns
